# A comparative evaluation of dimensional stability of three types of interocclusal recording materials-an in-vitro multi-centre study

**DOI:** 10.1186/1746-160X-8-27

**Published:** 2012-10-05

**Authors:** Sampath Kumar Tejo, Anil G Kumar, Vivekanand S Kattimani, Priti D Desai, Sandeep Nalla, Krishna Chaitanya K

**Affiliations:** 1 Principal Gandhi Dental College, Bubaneswar, India; 2 Department of Conservative and Endodontics, Saraswati Dhanwantari Dental College and Hospital, Parbhani, (MS), 431401, India; 3 Department of oral and maxillofacial Surgery, Saraswati Dhanwantari Dental College and Hospital, Parbhani, Pathri road, NH222, Parbhani, Maharashtra, India; 4 Department of Conservative and Endodontics, Gurunanak institute of dental science & research, Panihati, Kolkata, 114, India; 5 Department of Prosthodontics, SVS Institute of Dental Sciences, Mahaboobnagar, AP, India; 6 Department of Prosthodontics, Saraswati Dhanwantari Dental College and Hospital, Parbhani, Pathri road, NH222, Parbhani, Maharashtra, India; 7 Department of oral and maxillofacial Surgery, Saraswati Dhanwantari Dental College and Hospital, Parbhani, Pathri road, NH222, Parbhani, Maharashtra, India

**Keywords:** Polyether bite, Poly vinyl siloxane bite, Zinc oxide eugenol (ZOE) bite, Dimensional stability, Interocclusal records

## Abstract

**Background:**

The introduction of different interocclusal recording materials has put clinicians in dilemma that which material should be used in routine clinical practice for precise recording and transferring of accurate existing occlusal records for articulation of patient’s diagnostic or working casts in the fabrication of good satisfactory prosthesis. In the era of developing world of dentistry the different materials are introduced for interocclusal record with different brand names because of this; the utility of the material is confusing for successful delivery of prosthesis with lack of in vitro or in vivo studies which will predict the property of the material with utility recommendations.

**Purpose of the study:**

The aim of this multicenter research is to evaluate the time dependent linear dimensional stability of three types of interocclusal recording materials; which gives very clear idea to clinicians in regard to its usage in routine practice and recommendations for usage of the different materials. Also to find out ideal time for articulation of three types of interocclusal recording materials with accuracy.

**Materials and method:**

Commercially available and ADA approved Polyether bite registration paste (Ramitec), Poly vinyl siloxane bite registration paste (Jetbite) and Zinc oxide eugenol (ZOE) bite registration paste (Super bite) were used in the study.

A stainless steel die was made according to modified American dental Associations (ADA) specification no. 19. Each one of the tested materials were manipulated according to manufacturers’ instructions. The materials separated from die, 3-mins after their respective setting time, resulted in disks of standard diameter. Two parallel lines and three perpendicular lines reproduced on the surface. The distance between two parallel lines was measured at different time intervals i.e. 1 hour, 24, 48 and 72 hours by using travelling microscope (magnus) and compared with standard die measurements made according to ADA specification no.19 to find out the dimensional stability of these interocclusal recording materials. Total 120 samples were made for observation and results were subjected to statistical analysis. Statistical analysis was performed using analysis of variance (ANOVA) and then Tukey’s Honestly Significant Difference (HSD) test for comparison among groups at the 0.05 level of significance. After statistical analysis of the data, results were obtained and analyzed for interpretation.

**Results:**

The results shows significant difference between the dimensional stability of all three material at different intervals with p-value <0.05. Comparatively the polyether bite registration material showed less distortion with good dimensional stability compared to Poly vinyl siloxane bite (Jetbite), Zinc oxide eugenol(ZOE) bite (Super bite) at 1 hour, 24, 48, and 72 hours.

**Conclusion:**

The dimensional stability decreased with increase in time and is influenced by both material factor and time factor. Polyether was found to be more dimensionally stable interocclusal recording material, which was followed by Silicone and Zinc oxide eugenol (ZOE). The dimensional stability of Polyether was good. Zinc oxide eugenol is dimensionally more unstable when compared with polyether and polyvinyl siloxane. We recommend that the polyether interocclusal records must be articulated within 48 hours and Polyvinylsiloxane interocclusal records must be articulated within 24 hours and the ZOE should be articulated within 1 hour to get a correct restoration to have very minimum distortion and maximum satisfaction without failure of prosthesis.

## Introduction

The interocclusal record by various materials and methods will play a positive role in securing the desired occlusion in the fabrication of prostheses. Phillip pfaff in 1756 made the first interocclusal records using natural waxes [1-3]. Since then many materials and techniques have been evolved for recording interocclusal relationship. These materials are basically impression materials that have been modified to give better handling characteristics. These include impression plaster, Dental waxes, ZOE impression paste, acrylic resin, hydrocollides and newer one includes polyether and polyvinylsiloxane bite registration materials [2-9]. Since beginning of era of dentistry, the dental waxes and ZOE impression pastes are used till today as bite registration materials because of ease of manipulation and economically viable, as they are less time consuming and less skill dependent [5-7]. They have been adjudged as base materials for the development of newer materials and methods for recording interocclusal relations of teeth. Hence here it is used as control and comparative material with newly developed bite registration material.

Precise articulation of patient’s diagnostic or working casts is a prerequisite for fabrication of prosthesis [1]. A clinically acceptable prosthesis should be in harmony with the existing stomatognathic system. Recording and transferring of accurate existing occlusal records is of prime importance for a successful restoration. Interocclusal recording of the relationship of the mandible to the maxilla is a simple but complex procedure. The inaccuracies attributed to the interocclusal records can be divided into three categories [8]: 1) The biologic characteristics of stomatognathic system, 2) Manipulation of the material, and 3) The properties of the interocclusal recording materials.

Manipulation of the material such as variations in the manner with which the dentist manages the patient and the material during the clinical phases. Of all the properties of the interocclusal recording materials, most important is the dimensional changes caused due to delay in carrying materials to distant laboratories or delay in articulation or remounting of casts play a key role. To solve all these problems we require an ideal interocclusal recording material which is dimensionally stable with passage of time.

Interocclusal recording materials like wax and zinc oxide eugenol are used since beginning. But introduction of newer elastomeric materials in market has put clinicians in dilemma for the selection and usage. These elastomeric materials are chemically similar to the impression materials that have been used for many years [9-14]. Modifications have been made by adding plasticizers and catalyst to provide different handling characteristics [2]. However, it remains unknown whether these modifications in the parent impression materials have altered their properties like dimensional stability [1]. Delayed articulation of patients casts can occur for various reasons [10-18] therefore, the dimensional stability of interocclusal recording materials is most important in multi setup as all the factors has to be considered and analyzed for the recommendation or conclusion.

The ideal requirements of materials to be used for bite registration are[1-3,5-7] 1)It should become rigid and exhibit minimal dimensional change after setting 2) It should have limited resistance before setting to avoid displacement of teeth or mandible during closure 3) It should produce accurate record of incisal and occlusal surface of the teeth 4) It should be easy to manipulate 5) It should not produce adverse effects on the tissues involved in procedure 6) It should allow easy verification.

An ideal material for interocclusal record allows the intraoral placement of restorations without extensive adjustments. In order to achieve this goal the use of interocclusal recording materials which are dimensionally stable is of paramount importance. Much work has not been done to judge the dimensional stability; which is needed over a period of time. So multicenter research has been done, to evaluate the time dependent linear dimensional stability of three types of interocclusal recording materials; which gives very clear idea to clinicians with regard to its usage in routine practice and also recommendations for usage of the different materials.

## Materials and method

The following interocclusal recording materials were selected for the study;

1) Polyether bite registration paste (Ramitec, 3 M ESPE, AG Dental Products, Germany).

2) Vinylpolysiloxane bite registration cream (Jet bite, Coltene Whaledent, swezerland).

3) Zinc–oxide eugenol bite registration paste. (Super bite, Bosworth Company, Skokie).

### Equipment

**Figure 1:**
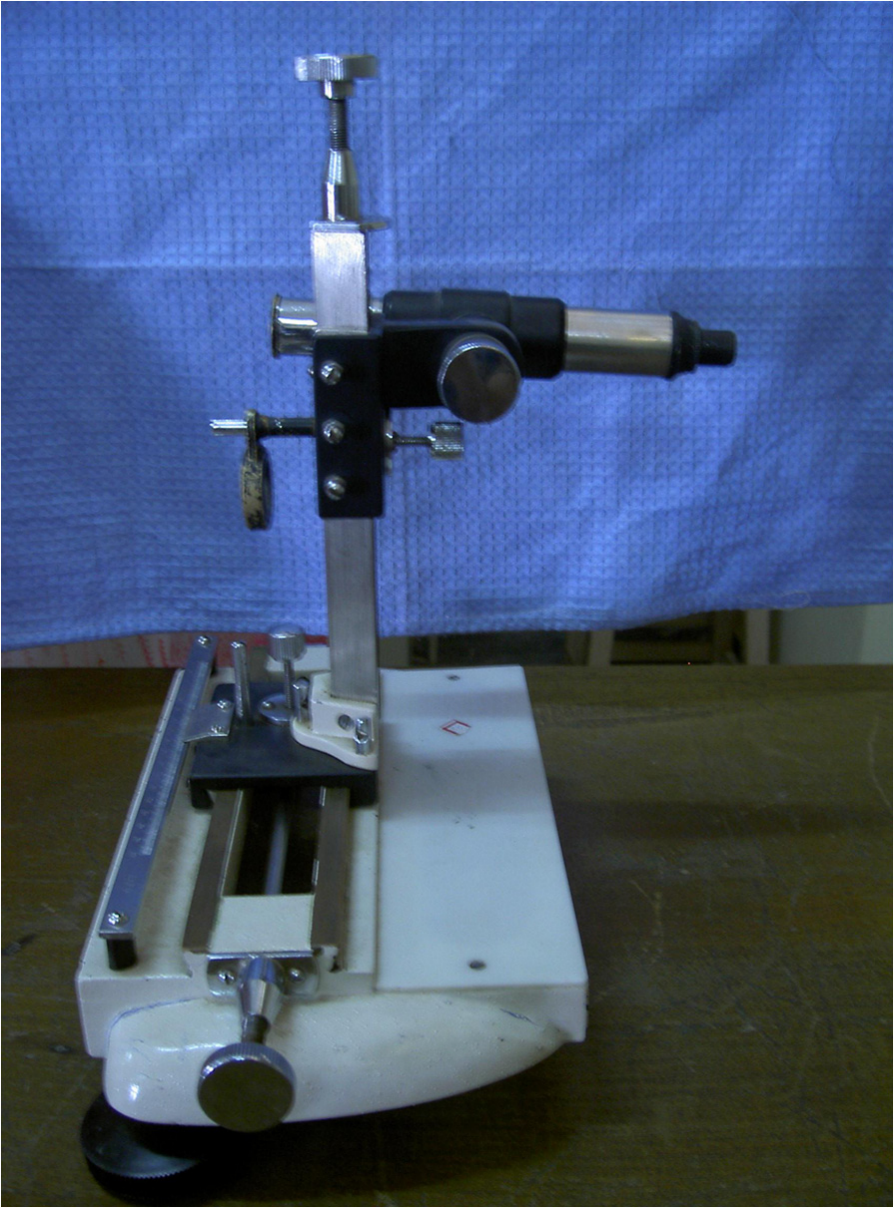
Travelling microscope.

1. Travelling microscope- (Figure 1).(Magnus, India)

2. Thermostat controlled water bath unit.

Methods followed in this study have been discussed under the following headings:

The method chosen to compare the dimensional stability was as per the testing methodology of ADA specification no.19-for elastomeric impression materials. 1) Preparation of mold, 2) Selection and manipulation of materials, 3) Preparation of samples, 4) Measurement of the distance between lines for determining dimensional stability.

### Preparation of mold

**Figure 2:**
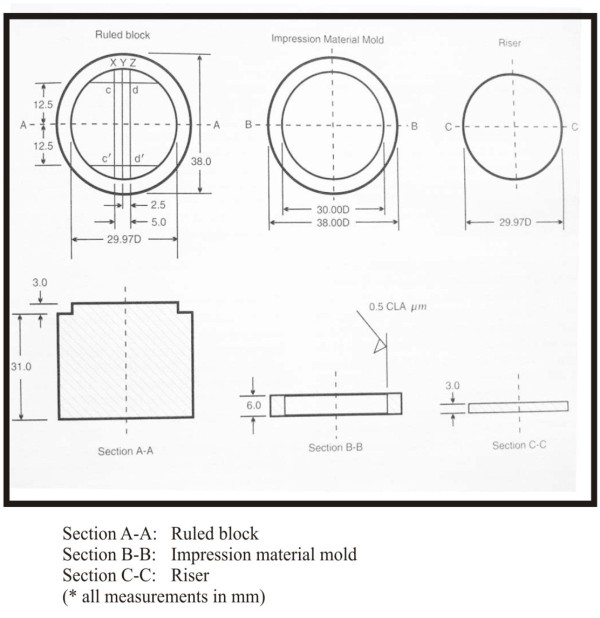
**Schematic diagram of blockreproduction.**

**Figure 3:**
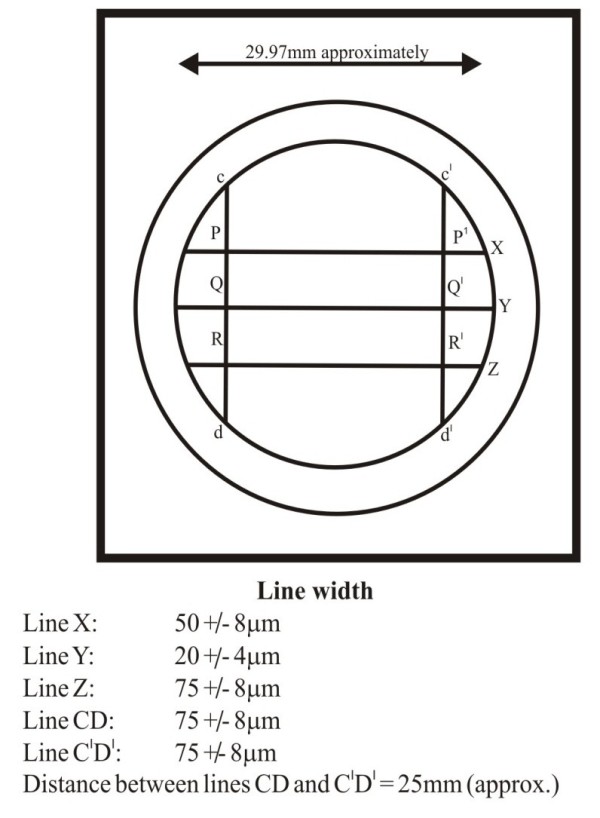
**Schematic diagram showing ruledsurface of die.**

**Figure 4:**
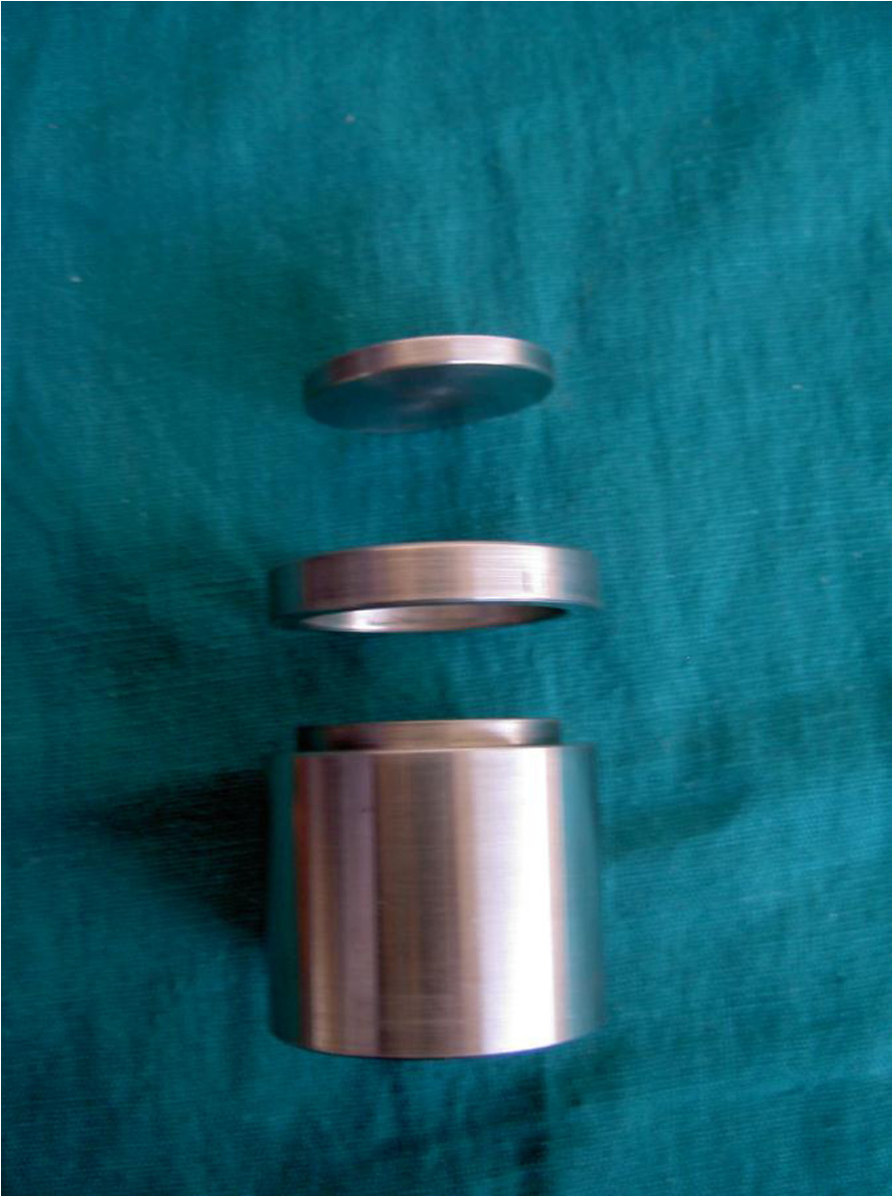
Master die with raiser.

A study mold (Figure 2, Figure 3, Figure 4) was prepared according to revised American Dental Association specification no.19 for non-aqueous elastic dental impression materials. It consists of a ruled block (AA), test material mold (BB) and a riser (CC). All parts were made up of stainless steel. The ruled block was having three horizontal lines of different width; small Y-line (24 μm), medium X-line (57 μm) and a thick Z-line (83μm), and two vertical lines CD and C^I^ D^I^ of 82 μm each. The lines CD and C^I^ D^I^ were separated from each other by 25 mm approx. The test mold was a cylinder of inner diameter 30 mm and depth of 6 mm to place the bite registration material. The riser was a stainless disk of diameter 29.9 mm and thickness of 3 mm.

### Manipulation of polyether bite registration paste

For Polyether bite (Ramitec E.S.P.E) required amount of equal lengths of pastes were dispensed on the mixing pad provided by the manufacturer. These two pastes were mixed together with stainless steel mixing spatula for 45 to 50 seconds to get a homogenous streak free mix. The mix was then collected on the mixing spatula and loaded in a plastic syringe provided by the manufacturer. The material was then spread on the surface of the die by taking precautions not to incorporate any air bubbles and a glass plate covered with polyethylene sheet and a weight of 500 g was placed over it. The material was allowed to set for 4–5 min in thermostatically controlled water bath to simulate mouth condition.

### Manipulation of polyvinyl siloxane bite registration material

The Polyvinyl siloxane bite (Jet bite, coltene whaledent), was supplied in the form of cartridge containing base and accelerator paste. The cartridge along with mixing tip was attached to an auto-mixing gun. The material which expelled from the gun was uniformly spread over the surface of the die. A glass plate covered with polyethylene sheet was placed on the die over which a weight of 500 g was kept and allowed to set for 4–5 min in thermostatically controlled water bath to simulate mouth condition.

### Manipulation of zinc oxide eugenol bite registration paste

For Zinc oxide eugenol bite registration paste (Superbite), equal length of both base and catalyst pastes were dispensed on the cool glass slab. By using stainless steel mixing spatula, they were mixed in clockwise direction for 45 seconds to get a streak free homogenous mix. The material was then collected on the mixing spatula and by taking precautions to avoid incorporation of air bubbles; the mix was spread over the surface of the die. Subsequently a glass plate covered with polyethylene sheet and a weight of 500 g was placed over it and the material was allowed to set for 8 to 10 minutes in thermostatically controlled water bath to simulate mouth condition.

### Preparation of samples

The whole assembly was then submerged in water bath of temperature 36 ± 1^0^C resembling open mouth temperature. Each assembly remained in the bath for the setting time suggested by manufacturer, plus 3 min to ensure polymerization in case of elastomeric materials.

**Figure 5:**
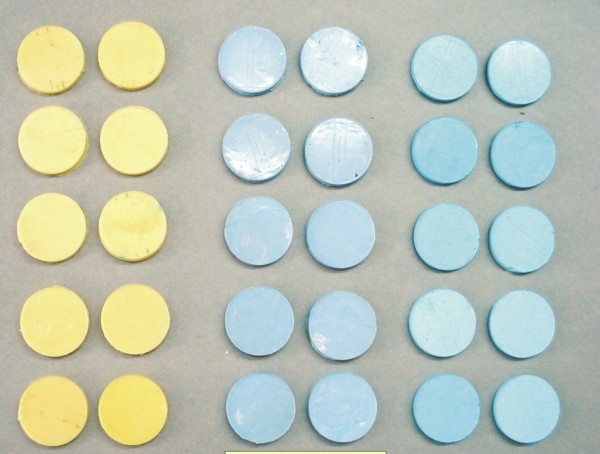
**Test samples-yellow polyether-blue middlecolumn ZnoE-blue Polyvinyl siloxane.**

After removal from the water bath, the material was separated from the die by using the disk (riser). The excess flash was trimmed using a Bard Parker knife. Thus prepared specimens (Figure 5) were measuring 30 mm in diameter, 3 mm in thickness and had the lines X, Y, Z, CD and C^I^ D^I^ lines on it. Similarly, all the 30 bite registration record samples from each center were obtained. In between days of observation, the samples were stored in a moisture free polyethylene bags at room temperature of 28 ± 2°C.

### Observation of samples for dimensional stability

The distance between the lines, CD and C^I^ D^I^, reproduced on the samples, was measured at three different points PP^I^, QQ^I^ and RR^I^ (i.e. at the intersections of these lines with the lines XYZ) by using travelling microscope with 10 X magnification. Three readings were obtained for each sample and the averages of these three values were noted. Likewise readings were made at different time intervals i.e.; 1 hour after removal of the material from the die, at 24 hours, at 48 hours and at 72 hours respectively for each of the samples. All the readings thus obtained were tabulated (Table 1) and subjected to statistical analysis for the comparison of dimensional stability of three interocclusal recording materials.

**Table 1:** **Showing the master chartof average data offour centers obtained duringstudy**

**Materials**	**sample no.**	**Dimensional stability : meandistance between lines (mm)**			
		**1Hour**	**24Hours**	**48Hours**	**72Hours**
**Polyether**	1	24.742	24.728	24.735	24.713
	2	24.791	24.79	24.756	24.74
	3	24.681	24.644	24.663	24.594
	4	24.749	24.731	24.69	24.704
	5	24.751	24.715	24.685	24.712
	6	24.775	24.761	24.741	24.671
	7	24.579	24.532	24.587	24.579
	8	24.729	24.676	24.682	24.662
	9	24.81	24.804	24.765	24.761
	10	24.795	24.779	24.761	24.774
**Silicone**	1	24.738	24.737	24.734	24.717
	2	24.728	24.728	24.702	24.682
	3	24.714	24.716	24.701	24.675
	4	24.746	24.749	24.737	24.706
	5	24.751	24.744	24.731	24.71
	6	24.753	24.749	24.726	24.711
	7	24.726	24.722	24.709	24.687
	8	24.766	24.753	24.735	24.703
	9	24.73	24.715	24.698	24.691
	10	24.735	24.725	24.699	24.668
**Zinc oxide eugenol**	1	24.704	24.644	24.638	24.612
	2	24.71	24.666	24.64	24.608
	3	24.706	24.663	24.649	24.622
	4	24.672	24.662	24.613	24.599
	5	24.72	24.648	24.638	24.602
	6	24.68	24.641	24.567	24.566
	7	24.691	24.654	24.64	24.625
	8	24.722	24.671	24.635	24.617
	9	24.736	24.665	24.624	24.611
	10	24.781	24.649	24.655	24.627

### Evaluation of dimensional change

The change in the Dimension is calculated by using the formula

(1) $$Dimensional\;change\;\%=\left(X-Y\right)x\;100$$

Where **X** is the standard measurement (μm) of CD and C^I^ D^I^ in the Die.

**Y** is the observed measurement (μm) of CD and C^I^ D^I^ in the sample

Statistical analysis was performed using analysis of variance (ANOVA) and then Tukey’s Honestly Significant Difference (HSD) tests for comparison among groups at the 0.05 level of significance.

The force of 5.564 n (500 g external weight + 67 g glass plate) was applied during making of the interocclusal registration. This force was chosen because researchers showed that the force required to compensate initial resistance of interocclusal material to closure; varying between 0.5 n to 13.8 n [8]. During the interval period of the study, all the specimens were stored in sealed dry polyethylene bags at room temperature (28 ± 2°C) as dimensional changes of elastomers can be reduced by storage in a sealed dry container and at room temperature [10-17].

The linear dimensional change of the interocclusal recording materials over time was measured in this study. The time intervals used in the study were selected considering the time taken to carry interocclusal recording materials to distant laboratories or delay in articulation or remounting of the casts if required. These measurements provide an indication regarding the dimensional stability. Ten samples for each material were made using a die similar to ADA specification No.19 at four different centers in different geographic location. The die and the interocclusal record material assembly were placed in to the water bath of 32 ±10°C to stimulate the oral temperature. Specimens having 3 mm thickness were considered for measurement because the accuracy may vary with time intervals and at different thickness. Travelling microscope was chosen for the measurement as per the testing methodology for ADA specification No.19. The results were obtained and Statistical analysis was performed using analysis of variance (ANOVA) and Tukey’s Honestly Significant Difference (HSD) test for comparison among groups at the 0.05 level of significance (Tables 2, 3, 4). Group A (polyether) presented the least linear changes of all the material tested, at all-time intervals followed by group B (polyvinylsiloxane) and group C (zinc oxide eugenol-ZOE) respectively.

**Table 2:** **Showing the comparison ofthe distance between horizontallines at different timeintervals for three interocclusalrecording materials using ANOVA**

**Hours Materials**	**1Hours**	**24Hours**	**48Hours**	**72Hours**
	**Mean ± SD**	**Mean ± SD**	**Mean ± SD**	**Mean ± SD**
**Polyether**	**24.740 ± 0.068**	**24.716 ± 0.082**	**24.707 ± 0.056**	**24.691 ± 0.065**
**Silicone**	**24.739 ± 0.015**	**24.734 ± 0.014**	**24.717 ± 0.017**	**24.695 ± 0.017**
**ZnOE**	**24.705 ± 0.020**	**24.656 ± 0.010**	**24.626 ± 0.024**	**24.607 ± 0.017**
**ANOVA**	F = 0.93 p = 0.44, NS	F = 3.42 p < 0.05, S	F = 6.35 p < 0.05, S	F = 6.36 p < 0.05, S

p ≤ 0.05 -significant(S), p ≤ 0.001 -highly significant(HS), p > 0.05 -not significant(NS).

**Table 3:** **Showing the comparison ofdimensional stability between threeinterocclusal recording materials atdifferent hours by usingMann Whitney test**

**Hours Materials**	**1Hour**	**24Hour**	**48Hours**	**72Hours**
**Polyether- Silicone**	**NS**	**p = 0.51 (NS)**	**p = 0.58 (NS)**	p = 0.86 (NS)
**Polyether – ZnoE**	**NS**	**p = 0.048(NS)**	**p = 0.001 (S)**	p = 0.003, (S)
**Silicone – ZnoE**	NS	p = 0.000 < 0.001 (HS)	p = 0.000 < 0.001 (HS)	p = 0.000 p < 0.001 (HS)

p ≤ 0.05 -significant(S), p ≤ 0.001 -highly significant(HS), p > 0.05 -not significant(NS).

**Table 4:** **Showing the comparison ofdimensional stability of threeinterocclusal recording materials atdifferent hours using ANOVA**

**Materials Hours**	**Polyether Mean ± SD**	**Silicone Mean ± SD**	**ZnOE Mean ± SD**
**1Hour**	**24.740 ± 0.068**	**24.739 ± 0.015**	24.705 ± 0.020
**24Hour**	**24.716 ± 0.082**	**24.734 ± 0.014**	24.656 ± 0.010
**48Hour**	**24.707 ± 0.056**	**24.717 ± 0.017**	24.626 ± 0.024
**72Hour**	**24.691 ± 0.065**	**24.695 ± 0.017**	24.607 ± 0.017
**ANOVA**	F = 0.911 p = 0.45 NS	F = 15.5 p < 0.05 S	F = 55.92, p < 0.05 S

p ≤ 0.05 -significant(S), p ≤ 0.001 -highly significant(HS), p > 0.05 -not significant(NS).

## Results

**Figure 6:**
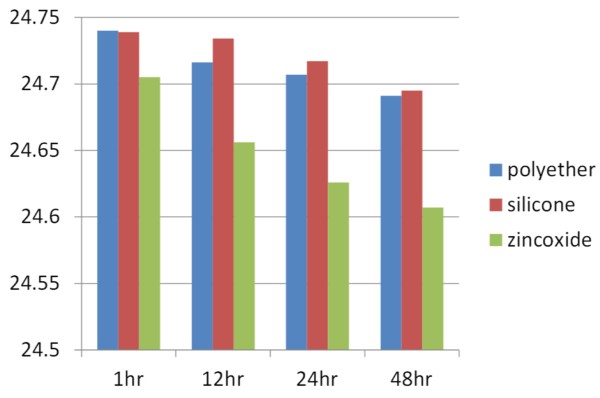
**Showing the comparison ofdimensional stability of threeinterocclusal recording materials atdifferent hours using ANOVA.**

The Figure-6 shows the comparison of dimensional stability of three interocclusal recording materials at different hours using ANOVA. The standard deviation (Table 1) with mean percentage of dimensional change for polyether at 1^st^ hour is 0.068 (0.011%), at 24 hours is 0.082(0.0125%), at 48 hours is 0.56(0.0127%) and at 72 hours is 0.65 (0.0133%) whereas polyvinylsiloxane at 1^st^ hour 0.015(0.012%), at 24 hours is 0.014(0.014%), at 48 hours is 1.017(0.015%) and at 72 hours is 1.017(0.016%) and Zincoxide eugenol at 1^st^ hour is 0.020(0.012%), at 24 hours is 0.010 (0.015%), at 48 hours is 0.024(0.018%) and at 72 hours is 1.017 (0.020%) indicating the dimensional stability of polyether is better than other two materials. The results shows (Table 2, Table 3, and Table 4 ) significant difference between the dimensional stability of all three material at different intervals with p-value <0.05. Comparatively the polyether bite registration material showed less distortion with good dimensional stability compared to Poly vinyl siloxane bite (Jetbite), Zinc oxide eugenol(ZOE) bite (Super bite) at 1 hour, 24, 48, and 72 hours.

## Discussion

The introduction of different interocclusal recording materials has put clinicians in dilemma that which material should be used in routine clinical practice for precise recording and transferring of accurate existing occlusal records for articulation of patient’s diagnostic or working casts in the fabrication of good satisfactory prosthesis.

Hence, the present in-vitro study was planned to compare the time dependent dimensional stability of three interocclusal recording materials at 1 hour, 24, 42 and 72 hours with commonly used ZOE bite registration material in multicenter with wide area of geography with different level of experience in the field of dentistry to reduce the bias. The geography will play important role in its transport, storage and even in manipulation of the same material at different temperatures in true clinical situation in the Indian scenario. The operator skill is paramount in the manipulation of material as the other faculty of dentistry leaving prosthodontics also involved in this study to know the variations in the skill for manipulation of material for its usage. Dimensional stability can be studied in all the three planes by using equipment’s like condymeter [8], computer axiotran, buhnergraph [10] and hydro-optic test measurement system.

Zincoxide eugenol is a traditional bite registration paste which is being used for a long time and has gained wide acceptance as impression as well as bite registration material because of its ease of manipulation and economy [19-25]. In this study ZOE expressed more dimensional change with passage of time i.e. 0.016% and continued to show drastic change over a period of 72 hours 1.017% . The results of this study seem to be in accordance with an experimental study by Michalakis K X, Pissiotis A, Anastasiadou [3] on physical properties of interocclusal recording media. ZOE undergoes continues contraction after 1 hour and continues to show significant dimensional change along with weight loss. This could be explained by fact that, the setting reaction of ZOE is basically acid base reaction resulting in salt formation [2,20-29]. Water formed during chelation reaction evaporates leading to weight loss and contraction over a period of time. However, the Balthazar - Hart Y et al. [26] states that eugenol free zinc oxide paste showed less dimensional change when compared to that of the eugenol. Other factors like immersion of these in different disinfectant solutions have been studied by many with changes in linear dimensional stability [28-33].

The results of polyvinyl Siloxane have shown significant change in dimensional stability after 24 hours (0.15%). This result is in accordance with the finding of Michalakis K et al. [3] were dimensional stability of elastomeric materials showed 0.18% dimensional change after 24 hours and significant change in dimensional stability even after 168 hours. The reason might be that polyvinyl siloxane has longer polymerization period resulting in sustained contraction period. That silicone bite registration paste undergoes sustained contraction even after 72 hours (0.016%).

In our study Polyether showed a 0.0125% dimensional change at 24 hours and with passage of time there is no much significant dimensional change ie 0.0127% on 48 hours. Change in the dimension was within the group of the ADA specification no.19 suggested value of 0.5% at 24 hours. Polyether bite registration is getting popular because of its dimensional stability. Our results are in accordance with Yvonne Balthazar, Sandrik J. [26], and Malone W.F.P, which showed that polyether exhibited no dimensional change after 24 hours [12]. Craig and peyton explains that Polyether sets by polymerization reaction so there will be volumetric shrinkage of the material during the reaction of about 0.3% [2,8,19,34].

## Conclusions

It is mandatory to choose a material not only depending on the clinical situation but also based on the time taken for the articulation. The dimensional stability decreased with increase in time and is influenced by both material factor and time factor. Polyether was found to be more dimensionally stable interocclusal recording material, which was followed by Silicone and Zinc oxide eugenol. The dimensional stability of Polyether is good. Zinc oxide eugenol is dimensionally more unstable when compared with polyether and polyvinyl siloxane. Even the cost effectiveness and superiority of quality of material and its ease of manipulation is very important factor which governs the selection of the material. We recommend that the polyether interocclusal records must be articulated within 48 hours and Polyvinylsiloxane interocclusal records must be articulated within 24 hours and the ZOE should be articulated within 1-hour to get a correct restoration to have very minimum distortion and maximum satisfaction without failure of prosthesis.

## Competing interests

The authors declare that they have no competing interests.

## Authors’ contributions

VSK, KCK and TSKB conceived of the study, and participated in its design and coordination and helped to draft the manuscript. The multicenter study carried out and reporting done by (1) VSK& KCK, (2) TSKB (3) PDD, and (4) AKG with SN. The compilation of article was done by AKG and VSK with literature search and review done by TSKB & PDD. The interpretation of data and analysis was carried out by SN, VSK and KCK. All authors read and approved the final manuscript.
